# Identification of plant-parasitic nematode genera in turfgrass using deep learning algorithms

**DOI:** 10.1038/s41598-025-29467-4

**Published:** 2025-12-07

**Authors:** Vikram Rangarajan, Fereshteh Shahoveisi, Benjamin D. Waldo, Sadegh Jafari

**Affiliations:** 1 Department of Computer Science, University of Maryland, College Park, MD USA; 2 Department of Plant Sciences and Landscape Architecture, University of Maryland, College Park, MD USA; 3 Mycology and Nematology Genetic Diversity and Biology Laboratory, USDA, ARS, Northeast Area, 10300 Baltimore Ave, Beltsville, MD USA; 4 Department of Computer Science, Iowa State University, Ames, IA USA

**Keywords:** Bayesian optimization, Convolutional neural network, Hyperband algorithm, Plant sciences, Machine learning, Pathogens

## Abstract

Plant-parasitic nematodes are an important threat to turfgrass. Left unmanaged, they can cause serious reductions in the quality and playability of golf courses and sports fields. Effective nematode management depends on accurate identification of the nematode genera extracted from soil samples. However, this process requires specialized expertise in nematology, which is often limited in plant diagnostic laboratories. Recent advancements in deep learning models offer promising solutions for the future of nematode identification. In this study, we evaluated the performance of EfficientNet V2-S, MobileNetV3-L, ResNet101, and Swin Transformer V2-B convolutional neural network model architectures in the classification of seven nematode taxa associated with turfgrass. Models were trained using a dataset of 5406 plant-parasitic nematode images where the dataset was split into 70, 15, and 15% for training, testing, and validation, respectively. Data augmentation and hyperparameter optimization using a combined Bayesian optimization and Hyperband algorithm (BOHB) approach were used to improve the model performance. Balanced classification accuracy on the test set was highest for EfficientNet V2-S and Swin Transformer V2-B at 94.63% and 94.34%, respectively. MobileNetV3-L and ResNet101 had lower balanced accuracies of 90.83% and 86.33%, respectively. Testing the models on an additional dataset using a user-end platform indicated the superiority of EfficientNet V2-S to other models with 82.47% accuracy. The findings of this study indicate the potential application of deep learning tools for accurate nematode identification to aid in diagnostics.

**Supplementary Information:**

The online version contains supplementary material available at 10.1038/s41598-025-29467-4.

## Introduction

Nematodes are non-segmented roundworms that belong to the phylum Nematoda. Most nematodes are free-living and contribute to beneficial ecological processes^1^. Other groups form parasitic associations and are economically important for animal and crop health^2,3^. Plant-parasitic nematodes are an important group of parasitic nematodes that feed on plants and cause an estimated 12–20% annual reduction in crop yields in the US^4,5^. Plant-parasitic nematodes are small, ranging 0.3–5 mm in length, and possess a needle-like stylet that is used by the nematode to pierce plant root cells and ingest the cell contents^6^. Heavy nematode feeding pressure can impair the ability of plants to take up water and nutrients and make the host more vulnerable to environmental stress and other pathogens^7,8^.

Turfgrass is a valuable agricultural crop worth approximately $60 billion in the US and is susceptible to plant-parasitic nematode feeding^9–11^. Common aboveground symptoms of nematode feeding include patchy areas of wilted, chlorotic, declining, or dead turfgrass^12^. Any reduction in visual quality is undesirable in highly maintained playing surfaces such as golf course putting greens. Determination of plant-parasitic nematode feeding injury based solely on above ground symptoms is not reliable since these symptoms can be caused by other stress inducing biotic and abiotic factors^13^. Diagnosis of plant-parasitic nematodes is performed by specialized diagnostic nematology laboratories. Nematodes are extracted from soil samples and a technical expert identifies and quantifies the plant-parasitic nematodes with the aid of a microscope. Even for experienced specialists, the plant-parasitic nematode identification and quantification process is tedious and time consuming. Training opportunities to develop plant-parasitic nematode identification skills are very limited and it can take years to gain the necessary experience to become a technical expert^14,15^. Therefore, tools that aid in nematode identification are highly desirable to increase efficiency for rapid plant-parasitic nematode diagnostics and broaden the availability of nematode diagnostics to laboratories lacking specialists.

Deep learning has recently received special attention among nematologists as robust and reliable models can achieve fast and accurate identification or counting of plant-parasitic nematodes^16–19^. Convolutional neural networks (CNN) are among the widely used architectures for nematode identification. Models such as fast R-CNN, ResNet, Xception, Pre-trained XGBoost, and YOLO models have shown a range of accuracy between 69 and 96% in nematode detection studies^17,20–22^. A study conducted by Pun et al.^23^ showed that YOLOv5, with a precision of 0.992, could successfully detect root-knot nematode eggs. Similarly, Agarwal, et al.^17^ reported YOLOv5 Baseline as the outperforming model among four other tested models such as EfficientDet and ResNet50, for plant-parasitic nematode detection where the mean average precision was 0.787 and the maximum precision was 0.836. Abade et al.^16^ evaluated the classification accuracy of several CNN models on an image dataset of five nematode genera. Among tested models, a customized model, NemaNet, with an accuracy of 96.76% outperformed other models such as InceptionV3, Xception, ResNet50, and EfficientNetB0. Some other models such as the Swin Transformer have been tested in detecting plant diseases such as cucumber and banana diseases^24^ however, its performance has not been tested in plant-parasitic nematode identification. Further research is required to evaluate the capability of such models in the field of nematology.

Some CNN nematode image classification studies used annotation of targeted morphological characters or morphometrics to identify taxa^17,21,22,25^. Other studies used images of entire adult or juvenile stages without morphological character annotation^16,20,26^. While both methods provide valuable information, annotations in supervised learning (i.e., labeling the data) provide key advantages in model training. Annotation enables the model to focus on relevant areas and enhance learning, and therefore, results in high-quality feature extraction^27^. Additionally, it improves performance in complex tasks by helping the model understand detailed patterns, leading to greater accuracy. Annotation can also reduce model error and enhance reliability and robustness, as training on well-labeled data results in fewer false positives and negatives^28^.

The vast majority of the image datasets used in CNN nematode image classification studies are collected from fixed specimens placed on microscope slides using compound microscopes. Diagnostic samples are often examined with an inverted microscope with specimens contained in a water suspension with a mixture of live and dead individuals. Live specimens actively move and can achieve variable positions that are not observed in dead specimens. Fixed specimens become rigid and the body shape can shrink or become distorted during the fixation process^29,30^. Data augmentation methods can be used to address the diversity of image capturing conditions such as light intensity, the orientation of a specimen, and image clarity^31^. In general, data augmentation is a valuable technique that offers several benefits in supervised learning. It enhances model generalization^32^ by reducing overfitting and results in good model performance on new and real-world data. Augmentation also increases data diversity^33^, which is especially valuable for limited or imbalanced datasets. It also improves model robustness, helping the model handle variations in real-world data more effectively. While augmentation offers several advantages, its application on fixed specimens may not replicate the complexity of body positions seen in live specimens. In nematology, using a dataset with images captured with inverted microscopes as well as compound light microscopes could provide more diverse and reliable input data for model training.

While CNN algorithms have been tested in the identification of nematodes affecting agricultural and vegetable host crops^16,17,25^, there is a gap in developing reliable models for the identification of nematodes affecting turfgrass. The purpose of this study was to create a novel dataset of nematode images for training of deep learning models EfficientNetV2-S, MobileNetV3-L, ResNet101, and Swin V2-B to identify seven classes of plant-parasitic nematodes associated with turfgrass. Diverse images were captured at 10×, 20×, and 40× objective magnifications and investigation of advanced hyperparameter tuning techniques was explored using a combined Bayesian optimization and hyperband algorithm (BOHB). This study helps advance machine learning approaches used for nematode identification by evaluating recent deep learning models on a unique turfgrass nematode dataset and to the best of our knowledge, used BOHB for hyperparameter and augmentation optimization for the first time in nematode classification.

## Results

### Nematode image dataset I

The original video dataset contained 1887 videos. After extracting every 100 frames from each video, a total of 31,684 images were obtained. In the next step, duplicate images were removed and automatic cropping was applied. Finally, a total of 5406 unique, cropped images of seven genera were obtained. The resulting images were grouped in dataset I and contained images of 829 *Helicotylenchus*, 997 *Hoplolaimus*, 434 *Meloidogyne*, 547 *Mesocriconema*, 666 *Pratylenchus*, 507 *Trichodorus*, and 1426 *Tylenchorhynchus*. Across microscope objective levels, the dataset consisted of 1644 10× images, 632 20× images, and 3130 40× images. Images that contained morphological features relevant for identification that were in focus, especially head and tail regions as well as the entire nematode body were selected for inclusion in dataset I. Blurry or out of focus images were not completely excluded to increase the variability of the dataset.

### Model performance

EfficientNetV2-Small architecture (EfficientNetV2-S) was the top performer in the classification of seven nematode taxa with a balanced accuracy of 94.63%, and Swin Transformer V2 (Swin V2-B) was next with an accuracy of 94.34% (Table 1). MobileNetV3-Large (MobileNetV3-L) achieved a balanced accuracy of 90.83%, while ResNet101 had the lowest balanced accuracy of 86.33%.

Macro precision and macro F1-score metrics also indicated that EfficientNetV2-S and Swin V2-B outperformed other models. When considering the macro one versus one receiver operating characteristic - area under the curve (OVO ROC-AUC) score, EfficientNetV2-S and Swin V2-B reached the highest values of 99.74% and 99.54%, whereas MobileNetV3-L and ResNet101 both had slightly lower values of 99.25% and 98.80%. EfficientNetV2-S and Swin V2-B had a specificity of 99.07% and 99.08%, respectively, showing a consistent ability to identify true negatives. MobileNetV3-L and ResNet101 followed closely with specificities of 98.35% and 97.59%. Additional training and validation metrics from dataset I are presented in Table S1.

**Table 1:** Statistical fitness metrics (%) of EfficientNetV2-S, MobileNetV3-L, ResNet101, and Swin V2-B on the test dataset.

Model	Balanced accuracy	Macro precision	Macro OVO ROC-AUC score	Macro F1-score	Macro true negative rate/specificity
EfficientNetV2-S	94.63	94.86	99.74	94.66	99.07
MobileNetV3-L	90.83	91.32	99.25	91.01	98.35
ResNet101	86.33	85.49	98.80	85.72	97.59
Swin V2-B	94.34	93.92	99.54	94.13	99.08

OVO ROC-AUC = one versus one receiver operating characteristic-area under the curve.

**Fig. 1:**
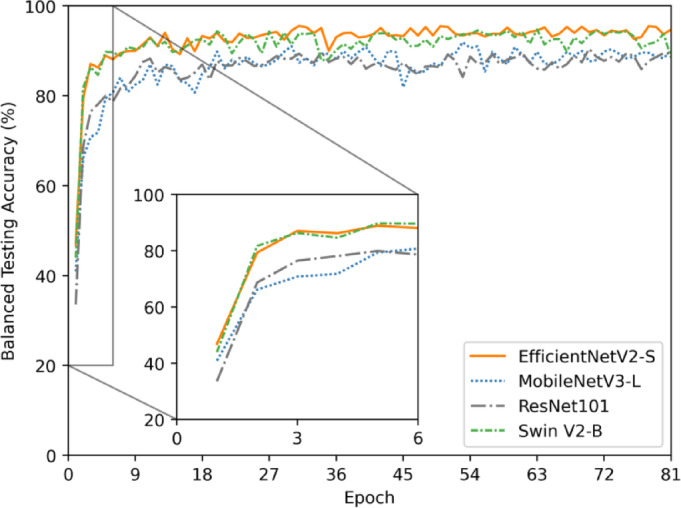
Balanced accuracy of the test dataset after every epoch of training for EfficientNetV2-S, MobileNetV3-L, ResNet101, and Swin V2-B models. Epochs 1–6 have been zoomed in to clarify the speed of convergence for each model.

Plotting the balanced test accuracy across epochs highlighted the performance of models in terms of convergence and stability (Fig. 1). After the first epoch, EfficientNetV2-S achieved the highest balanced test accuracy. EfficientNetV2-S exceeded 80% balanced testing accuracy with 87.10% at epoch 3, and Swin V2-B exceeded the 80% threshold achieving 81.74% balanced testing accuracy at epoch 2. Both models stabilize before epoch 20. In contrast, MobileNetV3-L and ResNet101 exhibited slower convergence with MobileNetV3-L achieving 80.76% balanced testing accuracy after 6 epochs and ResNet101 achieving 81.61% after 7 epochs.

### Image augmentation and hyperparameter tuning

Hyperparameter selection using a technique of combining Bayesian Optimization (BO) and the Hyperband algorithm (HB), called BOHB, was used for hyperparameter optimization. Hyperparameters were selected for training based on the highest balanced validation accuracy based on optimal combination of augmentation techniques such as rotation and brightness adjustment as well as hyperparameter selection for learning rate, dropout rate, classification head size, and batch size (Table 2).

**Table 2:** Best performing hyperparameters for EfficientNetV2-S, MobileNetV3-L, ResNet101, and Swin V2-B according to the bayesian optimization and the hyperband algorithm (BOHB) algorithm.

Model	Learning rate	Dropout rate (%)	Maximum random rotation (°)	Maximum random brightness	Size of classification head FC layer	Batch size	Best epoch
EfficientNetV2-S	7.37 × 10^− 5^	4.61	12.77	0.0631	495	32	71
MobileNetV3-L	1.68 × 10^− 4^	25.97	18.09	0.0986	674	32	40
ResNet101	2.20 × 10^− 5^	17.71	16.57	0.0317	700	32	50
Swin V2-B	1.77 × 10^− 4^	25.09	12.44	0.0944	921	64	20

*FC* fully connected.

Each of the four tested models in this study reached the best performance with unique hyperparameter configurations tuned over multiple epochs. The EfficientNetV2-S model was configured with a learning rate of 7.37 × 10^−5^ and a dropout rate of 4.61%. Data augmentation included a maximum random rotation of 12.77° and a brightness adjustment factor of 0.0631. The fully connected layer of the classification head had 495 units and a batch size of 32 was used. The model reached optimal performance at epoch 71. The MobileNetV3-L model used a more aggressive training approach to prevent overfitting with a higher learning rate of 1.68 × 10^−4^ and a dropout rate of 25.97%. The data augmentation involved a greater maximum random rotation of 18.09 degrees and a brightness factor of 0.0986, allowing the adaptation to a broader range of variations. The classification head featured 674 units, and a batch size of 32 was used. The model reached the optimum performance at epoch 40. The ResNet101 model adopted a learning rate of 2.20 × 10^−5^ and a dropout rate of 17.71%. Data augmentation parameters included a rotation of 16.57º and a brightness adjustment of 0.0317. The classification head was set at 700 units and the batch size was 32. The best performance was observed at 50 epochs. The Swin V2 model was trained with a learning rate of 1.77 × 10^− 4^ and a dropout rate of 25.09%. It incorporated data augmentation settings with 12.44 degrees of rotation and a brightness adjustment factor of 0.0944. This model had the largest classification head, with 921 units, and used a larger batch size of 64. The model reached the optimal performance relatively quickly at epoch 20. To ensure a batch size of 64 was optimal for the Swin V2-B architecture, the model was retrained using the same hyperparameters, but with a batch size of 32 (Table S2). With a smaller batch size, the model balanced test accuracy dropped to 92.79% compared to 94.34% with a batch size of 64, indicating a proper batch size selection by the BOHB algorithm (Table 1, S3). Gradual unfreezing was used during every hyperparameter tuning trial for each model. All layers were unfrozen over five epochs and only started the gradual unfreezing process once the model achieved a balanced validation accuracy of 50% (Fig. 1).

### Dataset II validation

**Fig. 2:**
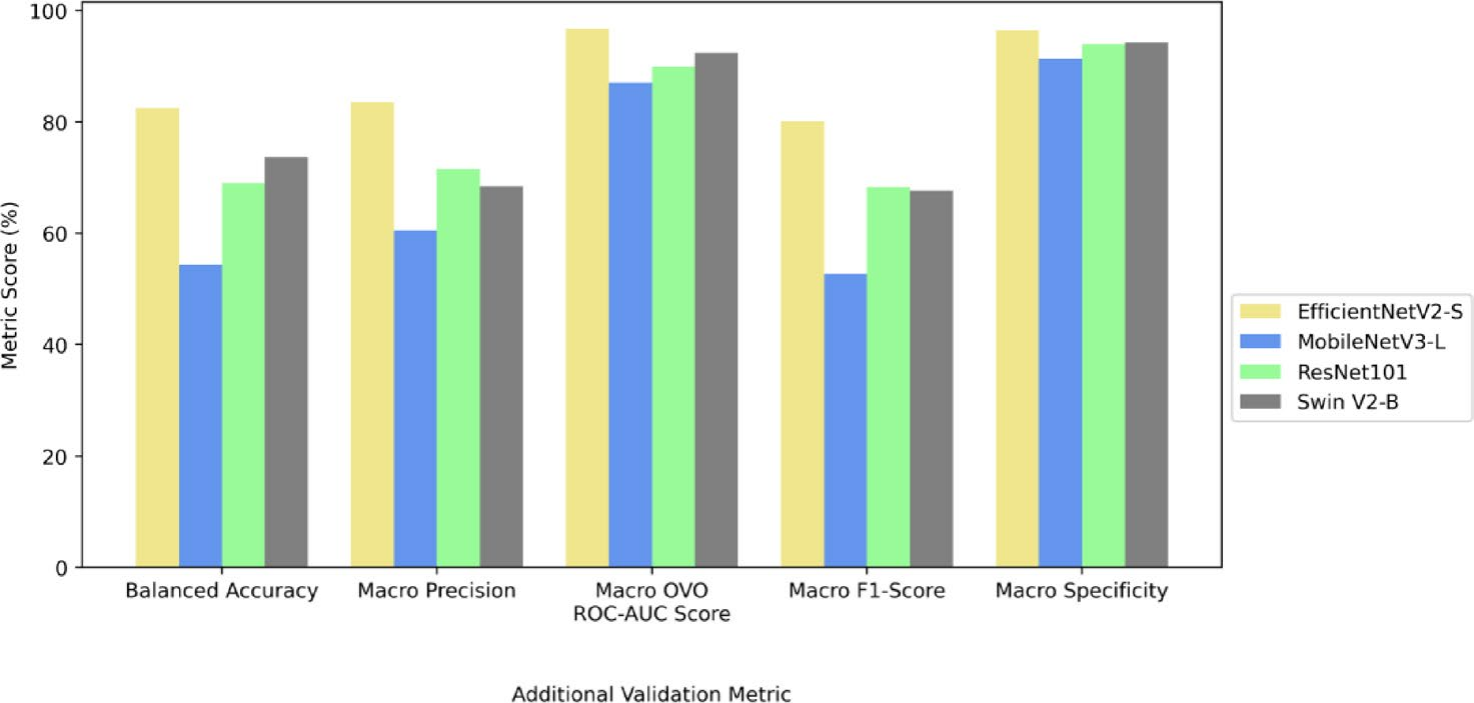
Metrics of EfficientNetV2-S, MobileNetV3-L, ResNet101, and Swin V2-B on the additional validation dataset.

We performed additional validation using a second dataset of 61 images to determine the generalizability of the models. The metrics mentioned in the model training were recorded to measure model performance on an end-user platform using dataset II as an additional validation dataset (Fig. 2). According to balanced accuracies, EfficientNetV2-S had 82.47% accuracy, followed by Swin V2-B with 73.68%, ResNet101 with 68.94%, and MobileNetV3-L with 54.35% (Table S4). Macro precision ranged between 60.45 and 83.56% for all models with EfficientNetV2-S indicating the highest precision. Macro receiver operating characteristic-area under the curve (ROC-AUC) scores ranged between 87.01 and 96.70%, and macro F1-scores ranged from 52.70 to 80.02%. The macro specificity reflected a similar trend, with EfficientNetV2-S leading with 96.55%, Swin V2-B at 94.25%, ResNet101 at 93.99%, and MobileNetV3-L at 91.37%.

**Fig. 3:**
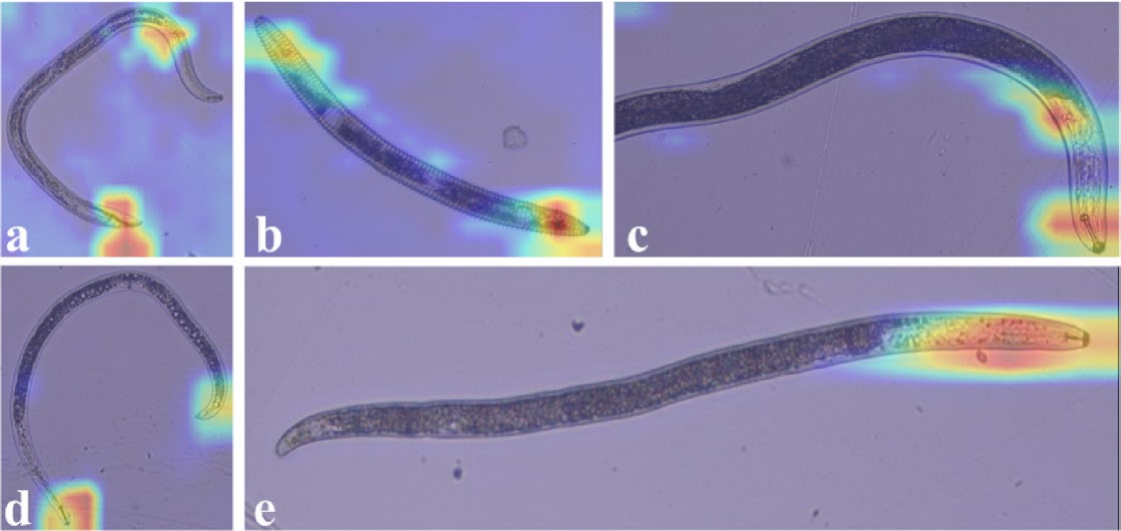
Examples of heat map images generated using the Gradient-weighted Class Activation Mapping (Grad-CAM) method on additional validation data in dataset II for the EfficientNetV2-S model. The red and blue regions highlight the most and the least discriminative regions, respectively.

The models were incorporated into the Gradio application hosted on Hugging Face Spaces which can be accessed at https://huggingface.co/spaces/VikramR/NematodeClassifier and is available for public use. This application allows for the classification of images containing a single nematode belonging to one of the 7 genera used by the model. In addition, the automatic cropping pipeline was included for testing with these new set of microscope images that were not cropped. Additionally, the Gradient-weighted Class Activation Mapping (Grad-CAM) technique was used to visualize sections of the images that EfficientNetV2-S mainly focuses on for classifying images^34^. Dataset II was used for the visualization. In Fig. 3, the red areas highlight the regions the model identifies as the most important for classification, while the blue areas represent the least relevant regions.

## Discussion

The image data used in this study targeted plant-parasitic nematode genera that impact turfgrass, distinguishing it from existing published datasets. For example, datasets by Abade et al.^16^ and Agarwal et al.^17^ focused on nematodes that broadly impact agricultural crops or crops other than turfgrass. This targeted approach is essential given the underrepresentation of turfgrass in nematology research and allows us to address the unique challenges faced in diagnosing nematodes affecting this crop. Incorporating images from both inverted and compound microscopes in this study resulted in the generation of a dataset representing the real-world diagnostic conditions more accurately. In contrast, other nematode image datasets such as those from Qing et al.^20^ and Shabrina et al.^31^ typically used only compound microscopes and fixed specimens, resulting in somewhat rigid body shapes that may not capture the live morphology of nematodes. The inclusion of images at 10×, 20×, and 40× magnifications in our dataset provided a range of visual scales, enhancing the model’s ability to generalize across different diagnostic needs. Unlike NemaNet’s dataset^16^ and the nematode egg dataset by Pun et al.^23^, which used only a single magnification level, our varied approach offers flexibility, supporting better model adaptability in various diagnostic scenarios^16,23^. Future work incorporating images obtained using additional optical setups could further enhance generalization of the models trained in this study. Building nematode image datasets for specific cropping systems is valuable not only for diagnostics for a specific crop, but also future work in training models for more comprehensive nematode identification across cropping systems. The datasets used to train models in our study contained a single individual nematode per image. Images that contain multiple nematodes belonging to different genera will likely not result in high accuracy predictions by the models. In future work, expanding the model training pipeline to incorporate datasets with multi-class images would likely improve applications in diagnostic settings. Our models would be most appropriate in settings where samples are diluted so that images may be obtained with one nematode specimen per image or where nematodes of interest are picked from samples and isolated for identification.

The results of this study indicated that EfficientNetV2-S architecture was the top performer in accuracy for the classification of seven nematode taxa followed closely by Swin V2-B. MobileNetV3-L was ranked third and ResNet101was ranked fourth. EfficientNet models have also shown encouraging results in nematode microscopic image classification. In a study conducted by Shabrina et al.^31^, EfficientNetV2M was reported as the top-performing model in the classification of 11 nematode genera, with an overall mean accuracy of 98.66%. The high efficacy of EfficientNet models in the identification of plant pests and diseases has been reported previously^35–37^. For instance, EfficientNet-B3 was 95.1% accurate in distinguishing peach diseases^38^, and EfficientNet-B5 was 94.85% accurate in the classification of cucumber diseases^39^. The capability of EfficientNet to recognize small features was assumed to be a contributing factor to its strong performance in the Wang et al.^39^ study, which could be advantageous for nematode identification studies when debris from environmental samples is present. In another study, EfficientNetV2-S resulted in a training accuracy of 97.5% in the classification of plant diseases in the PlantVillage-mixup dataset, where ViT-B and Inception V3 models were 81.72 and 89.91% accurate, respectively^40^.

Swin V2-B showed great promise in our study. Studies evaluating Swin models for nematode identification are minimal. One of the few reports is on the application of the model in the segmentation of *Caenorhabditis elegans* with an average precision of 0.99^41^. There are also a limited number of studies reporting the performance of the Swin models in plant disease classification. Wang et al.^39^ similarly reported high performance of Swin models with accuracies of 96.81% for the original Swin Transformer and 98.97% for the improved Swin Transformer in their cucumber disease classification study. Swin models work well at extracting features from images with complex backgrounds, which could prove useful in future work involving nematode samples that contain debris in the background. The performance of Swin models is normally exceptional, but high computational resource usage could be a downside for devices with constrained energy consumption. However, Swin may require less training time than other models like ResNet^42^.

Studies evaluating MobileNet al.gorithms for nematode identification are also limited. Agricultural studies that have utilized MobileNet for image classification have focused on foliar plant diseases. MobileNetV2 achieved a precision of 91% for recognizing foliar diseases^43^, which is very similar to the macro precision in our study. In a tomato leaf disease identification study, test accuracy for a fine-tuned MobileNetV3 model was 85%^44^. Chawla et al.^45^ evaluated several models for yellow vein mosaic virus symptom identification and found accuracy was 98.9% for MobileNet, 97.1% for EfficientNet, and 52.1% for ResNet50. ResNet101 was the lowest performing model in our study. ResNet models have been commonly tested in the classification of plant diseases and pests, but at times, it underperformed compared to other CNN model architectures. In nematode classification studies, Shabrina et al.^46^ compared 15 commonly used CNN models and found the mean class accuracy for ResNet50v2 to be 91.4% which was slightly lower than EfficientNetV2S at 94.8%. Angeline et al.^47^ used the ResNet101 model for the classification of free-living and plant-parasitic nematode images, where the model reached a top accuracy of 87.5%, very similar to ResNet101 accuracy in our study. Qing et al.^20^ reported 94–97% accuracy of ResNet101 which could identify 60% of genera in the training set. The small size of the training dataset was likely a contributing factor to the low recognition of genera. ResNet has been reported to more commonly mistake background noise for areas of interest, which can lead to underperformance relative to other models, such as EfficientNet and Swin that better distinguish background noise^39^.

EfficientNetV2-S achieved the highest balanced test accuracy, after the first epoch, indicating faster early learning and convergence compared to the other models. While all models had some fluctuations in performance, EfficientNetV2-S was the most stable model reflected in balanced test accuracy and showed consistently high accuracy throughout the training process. Both EfficientNetV2-S and Swin V2-B exhibited rapid convergence. The potential reason for less fluctuation of EfficientNetV2-S could be the model architecture, which employs a progressive learning strategy. This approach begins training with lower image resolutions and gradually increases them as training progresses^48^. As a result, this strategy facilitates smoother optimization and ensures more stable performance over time. Swin V2-B demonstrated a similar, though slightly less stable, performance across epochs. The Swin V2-B model has excellent stability because of the shifting window-based self-attention mechanism and hierarchical architecture. This architecture contributes to consistent performance across epochs by effectively capturing local and global information with little computational overhead^49^. In contrast, MobileNetV3-L and ResNet101 exhibited slower convergence. MobileNetV3-L showed significantly more fluctuations throughout the training, even after reaching higher accuracies. The lightweight and efficient design of MobileNetV3-L could be a contributing factor to the instability of the model by making it susceptible to changes in hyperparameters, which can result in performance fluctuations during training^50^. ResNet101, built with residual connections to support deeper architectures to manage vanishing gradients effectively. However, despite these connections, the depth of the model still leads to some instability, making it slightly less consistent than MobileNetV3-L^51^.

MobileNetV3-L, EfficientNetV2-S, and ResNet101 are all CNN-based models, whereas Swin V2 is based on the Vision Transformer (ViT) architecture. Swin V2-Base has 88 million parameters^49^, MobileNetV3-L has 5.4 million parameters^52^, EfficientNetV2-S has 24 million parameters^48^, and ResNet101 has 44.5 million parameters^53^. Models used in the study varied in size and performance. Swin V2-B, despite its larger architecture and higher computational requirements, such as a classification head with 921 neurons and a batch size of 64, performed similarly to the more computationally efficient model, EfficientNetV2-S. MobileNetV3-L is designed to be lightweight and efficient, however, this might result in lower accuracies, as observed in this study. ResNet101 requires substantially more processing power and memory because of its 101-layer depth, which is deeper than both MobileNetV3-L and EfficientNetV2-S. Although useful for understanding intricate patterns, this extra depth can cause overfitting when dataset size is constrained^53,54^, which could account for its lower accuracy. Also, ResNet101 depth results in slower training and inference times compared to the more lightweight MobileNetV3-L.

BOHB, has been reported as a promising technique in hyperparameter optimization^55,56^. Hyperparameter tuning is a crucial step in improving the performance of the CNN models. While some studies report model training using basic hyperparameter tuning without specialized optimization methods^16,17,37,46,47,57^, others use hyperparameter optimization techniques such as random search, BO, and the Asynchronous Successive Halving Algorithm^58,59^. Manual hyperparameter tuning is a time consuming process that requires expertise. Further, an effective hyperparameter tuning requires exploring many configurations which is inefficient due to the long training times for neural networks. Restrepo-Arias et al.^60^ reported BO as the best performing optimization technique to achieve better-trained artificial neural networks for the classification of plant diseases. HB has also been an effective technique for hyperparameter search in the field of plant disease detection and classification^61,62^. Combining the BO and HB techniques could improve the performance of the model to a greater extent as it benefits from the advantages of both techniques, including the strong anytime performance of HO with the strong eventual performance of BO^55,63^. In future nematode identification studies, implementing optimization algorithms like BOHB may help improve overall model performance by automating selection of the best combinations of hyperparameters. Image augmentation can enhance CNN performance by artificially expanding training datasets which improves model robustness, generalization, and accuracy, particularly in handling variations in image orientation, scale, and lighting^64^. Choosing the augmentation technique depends on factors such as dataset characteristics and model requirements. Application of various augmentation techniques in nematode classification and plant disease identification have been previously reported^16,21,31,65,66^. In a study conducted by Shabrina et al.^31^, the performance of augmentation techniques varied based on the optimizer and CNN model. For instance, for ResNetV2–101, Gaussian blur resulted in the highest accuracy when the Adaptive Moment Estimation (Adam) optimizer was used while normal images and Stochastic Gradient Descent (SGD) optimizer had the highest accuracy. Similarly in EfficientNetV2M, Gaussian noise and normal images resulted in the best performance of the models when Adam and SGD optimizers were used. While comparing the efficacy of different augmentation techniques was not the objective of the present study, hyperparameter optimization using the BOHB algorithm specified different optimum random brightness and random rotation for each model.

Gradual unfreezing was used during every hyperparameter tuning trial for each model. This method was originally introduced for natural language processing models (fast.ai) in the Universal Language Model Fine-tuning method^67^. The authors found that gradual unfreezing prevented catastrophic forgetting of important features which normally occurs when fine-tuning all model layers simultaneously. This method has since been applied in image processing tasks. For example, this technique was used in a tomato disease detection study and the model achieved an F1-score of 99.5%^68^. Gradual unfreezing has also been used in other fields of biology such as in salivary gland tumor detection, achieving an accuracy of 89% ^69^. Traditionally, gradual unfreezing involves unfreezing one layer per epoch^67^, but the technique was modified to unfreeze all layers over five epochs the gradual unfreezing process only started once the model achieved a balanced validation accuracy of 50%. A stable training process was observed in our study despite the modification. A notable observation was the selection of different batch sizes by the BOHB algorithm. It selected a batch size of 64 for the Swin V2-B model, while this value was 32 for all other models. This distinction can be attributed to the fact that Swin V2-B is a Vision Transformer model that employs shifted window attention, allowing it to apply localized attention mechanisms. This capability enables the model to handle larger batch sizes more efficiently. Moreover, larger batch sizes contribute to stabilizing the training process for transformer-based models. The use of BOHB in this study provided a clear advantage over manual selection approaches. By enabling efficient exploration of hyperparameters, BOHB offers a guided, iterative method that reduces resource use and has the potential to enhance model accuracy.

Additionally, Grad-CAM technique was used to visualize the portions of images that EfficientNetV2-S primarily focused on for identifying nematode classes from images^34^. The visualization performed using the additional validation dataset II in our study revealed that the model focused on different parts of the nematode body, including the head region (stylet and esophageal overlap) and tail. Morphological characters in these regions are used in dichotomous keys to differentiate the classes of genera included in the study^70^. Publicly deploying models trained on these datasets can provide a more user-friendly approach for interacting with models. Other studies also deployed models in web applications for demonstrations. Qing et al.^20^ developed NemaRec, a deep learning-based web application, for nematode identification and ecological indices calculations. The identification success rate was reported between 20 and 60% in that test version. In another study conducted by Shabrina et al.^31^, one of the best-performing models, EfficientNetV2B0, was incorporated into a cloud-based platform named Heroku. While we could not access the platform, the authors reported 97.94% identification accuracy for nematodes in perfect and undamaged conditions.

## Conclusions

The present study highlights the potential of deep learning models for nematode identification. EfficientNet V2-S was the top performing model in this study at 94.63% balanced classification accuracy on the test set and 82.47% balanced classification accuracy on the additional dataset II. BOHB was successfully implemented to select optimal data augmentation and hyperparameters to improve the model performance. The models developed in this study were optimized for single-taxon classification, but incorporating multi-class identification capabilities could greatly enhance their utility in diagnostics, especially in complex soil samples with diverse nematode populations. Additionally, validating the performance of our top-performing model, EfficientNet V2-S, across larger, more diverse datasets and user-end platforms will be essential to confirm its robustness in operational contexts. The overall findings of the study may contribute as a building block towards building a comprehensive nematode identification model and identification interface for nematode diagnostics in turfgrass.

## Materials and methods

### Nematode image dataset I

**Fig. 4:**
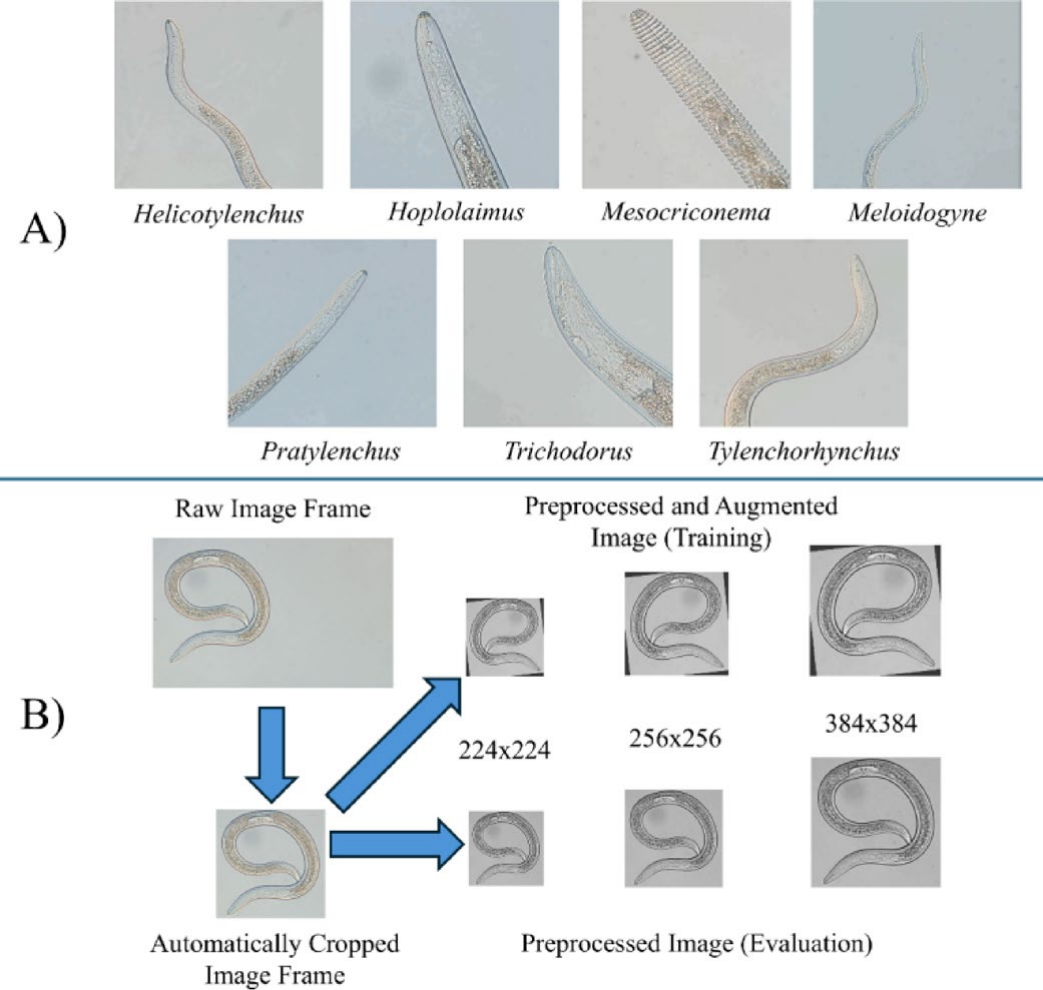
Examples of plant-parasitic nematode genera images used in the data analysis (**A**), and the image preprocessing pipeline (**B**). This pipeline automatically cropped image frames and then processed them into different image sizes (224 × 224 for MobileNetV3-L and ResNet101, 256 × 256 for Swin V2-B, and 384 × 384 for EfficientNetV2-S). The image pipeline preprocessed the image and only applied augmentation for model training.

Soil samples were collected in Maryland from golf course greens, athletic fields, and the USDA Beltsville Agricultural Research Center. Samples were collected with a 2.5-cm diameter soil probe and stored in polyethylene bags at 5 °C. Nematodes were extracted from soil using the conventional practice of sugar flotation centrifugation typical of many diagnostic laboratories^71^. Live adult and juvenile nematodes associated with turfgrass were picked from samples and grouped together by genus (Fig. 4A). Nematode genera commonly associated with cool-season turfgrass in Maryland and the Mid-Atlantic U.S. were selected according to a survey conducted by Shahoveisi and Waldo^72^, which included *Helicotylenchus*,* Hoplolaimus*,* Meloidogyne*,* Mesocriconema*,* Pratylenchus*,* Trichodorus*, and *Tylenchorhynchus.* Results of genera prevalence and abundance can be found in the published survey^72^.

Imaging was performed using an inverted microscope to replicate diagnostics microscopy conditions as well as a compound light microscope to capture greater morphological detail under higher magnification. Zeiss Observer Z1 inverted microscope (Carl Zeiss, Oberkochen, Germany) fitted with a Zeiss Axiocam 503 color camera was used. Nematode specimens were placed in a 6.5-cm diameter gridded petri dish filled with tap water and videos were recorded of specimens using 10× and 20× objectives and processed using Zen blue version 2.6 software (Zeiss). Olympus BX51 compound microscope (Olympus Corp., Tokyo, Japan) fitted with a DP74 Olympus camera was used. CellSens version 4.1 software was used to process videos (Olympus). Nematodes images were collected with the compound microscope using 10× and 40× objectives with specimens placed on a 1-mm thick microscope slide with an agar pad covered with a 0.15-mm thick circular coverslip^73^. Videos were collected of a mixture of mobile and non-mobile nematodes for image extraction from both the inverted microscope and compound microscope. The resolution of videos taken with the Zeiss camera was 1920 × 1452 pixels and Olympus videos were 1920 × 1200 pixels. The final videos were cropped to 1920 × 1080 pixels to standardize the dataset.

Dataset creation is described in detail in Algorithms 1–3 (Table S5). To convert the videos to an image dataset, images were extracted every 100 frames for each video using the Open Computer Vision (OpenCV) library^74^. Individual frames were extracted from each video using a customized pipeline. In the first step, an algorithm was used to extract the 100th frame out of the total number of frames in a video and edges of the frame were cropped to remove embedded scale bars. Frequently, frames extracted from the same video were nearly identical. Therefore, a second algorithm was implemented to remove duplicate frames. The extracted frames were sorted by extraction timestamp from each corresponding video. Each frame was cropped for the region of interest and a binary mask was generated marking where the nematode was present in the frame. The percentage overlap between consecutive frames was computed and frames with less than 80% overlap similarity, indicating nematode movement, were retained in the dataset.

In addition, the images required cropping to remove the background. For this purpose, binary images containing only black or white pixels outlining the nematode in the image were created. To achieve this, images were subjected to grayscale^75^, and then canny edge detection^76^, with the first and second thresholds set to 25 and an aperture size of 3, was applied to highlight the edges of the nematode. The images were grayscaled as a requirement to apply Canny edge detection and eventually find the bounding boxes around the nematode for cropping. However, the final cropped images were saved in color. To fill in gaps in the outline of the nematode where canny edge detection failed, dilation and then erosion^77^ from OpenCV with 11 × 11 kernels of ones were applied, each with three iterations. After this process, the output images contained the outline of the nematode with a completed contour. To remove other debris in the image, the largest contour (nematode body) was selected and cropped. To eliminate duplicate images from a given video, which is the result of slow-moving or dead nematodes, the binary images of nematodes in adjacent frames were compared using average pixel accuracy to determine if the nematode’s body position had shifted. After testing values from 75 to 95% with a 5% interval, the threshold to determine if the nematode shifted was set to 80%, meaning that binary image frames with an agreement of more than 80% are considered duplicates, while frames with less than 80% are considered unique. A higher threshold caused many similar image frames in the output dataset, while a lower threshold value pruned too many images, decreasing the dataset size. The remaining images were split according to the video group such that all images of nematodes that originated from the same specimen belong to only one of the training, validation, or test sets to ensure dataset independence. The dataset consisting of 5,406 images was split for training (~ 70%; 3,792 images), validation (~ 15%; 813 images), and testing (~ 15%; 801 images). Across microscope objective lenses, the training dataset consisted of 1,105 10× images, 436 20× images, and 2,251 40× images. The image dataset used for model training and model evaluation is referred to as dataset I. The image dataset can be accessed at 10.15482/USDA.ADC/27244674.

### Data preprocessing

The images varied in size because the automatic cropping did not resize the images to a standard shape; therefore, the images were resized according to the input size requirements and to ensure the compatibility of images with different models. The images were resized based on Torchvision defaults: 384 × 384 for EfficientNet, 224 × 224 for ResNet and MobileNet, and 256 × 256 for Swin Transformer models (https://pytorch.org/vision/stable/models.html*).* For all models, following resizing, the images were converted to 32-bit floating point numbers and scaled. The images were then normalized across the 3 RGB channels using Torchvision’s standard values using Torchvision Transforms v2. The normalization was centered around a mean of 0.485 on the red channel, 0.456 on the green channel, and 0.406 on the blue channel. The standard deviation was 0.229 on the red, 0.224 on the green, and 0.225 on the blue channels. The images were then converted to grayscale to ensure the background lighting color of the microscope did not contribute to the classification process. The selected Torchvision models require three channel inputs, so we used a three channel grayscale conversion. This preprocessing stage is applied during both model training and evaluation.

Data augmentation was performed on the training set to increase the diversity and generalization potential of the image dataset. Image manipulation techniques included random image rotation, random flips and 90° rotations, and random brightness^64^ provided by Torchvision transforms v2 (https://pytorch.org/vision/stable/transforms.html*)* and were applied after image preprocessing and before passing the images to the models. The image processing pipeline is shown in Fig. 4B.

### Model architecture and training

**Fig. 5:**
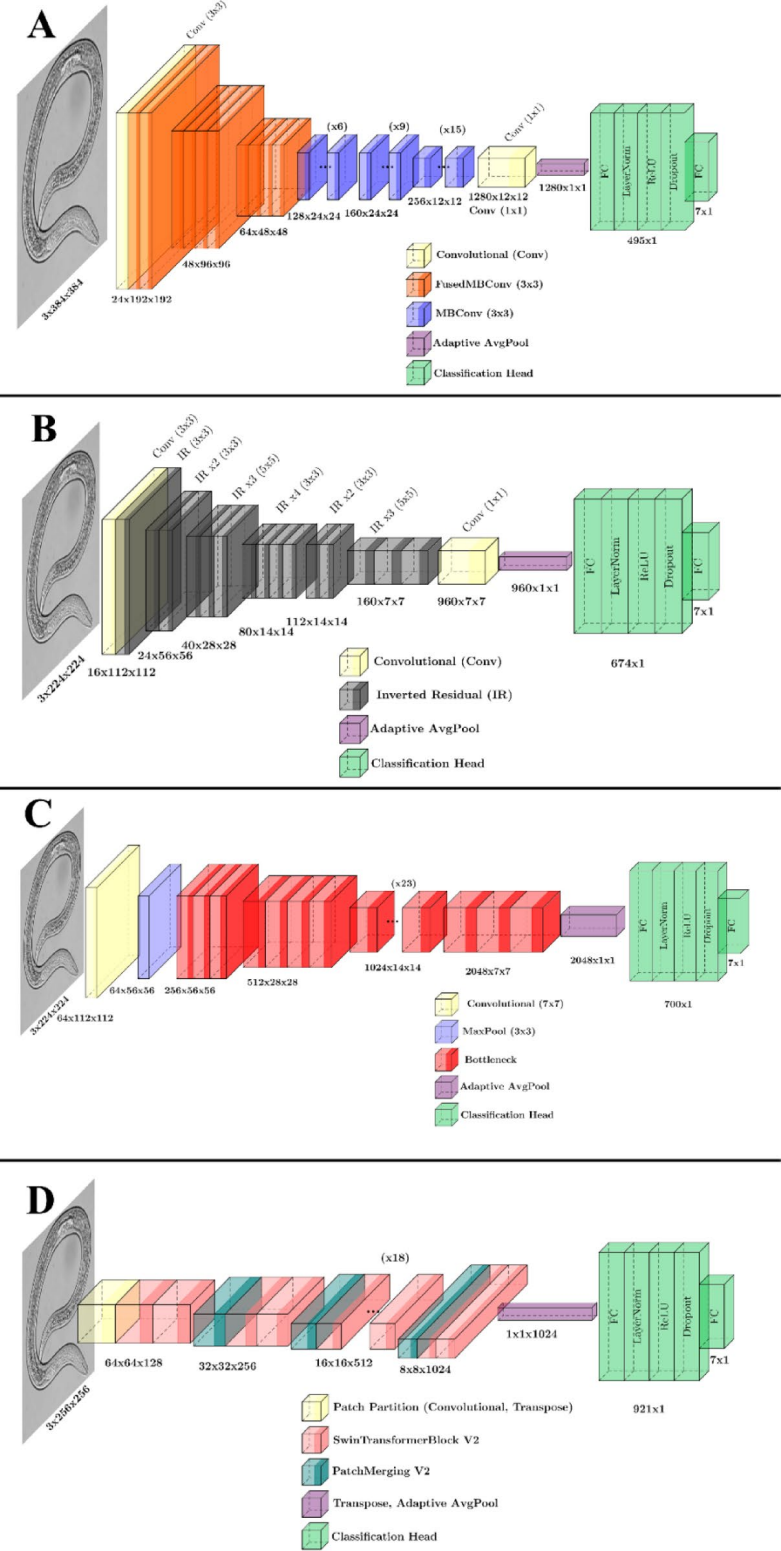
Architectures of fine-tuned models used in this study. **A** EfficientNetV2-S containing a total of 10 FusedMBConv modules followed by 30 MBConv modules with a sample input image size of 3 × 384 × 384. **B** MobileNetV3-L model used in this study contains a total of 15 inverted residual bottlenecks with a sample input image of size 3 × 224 × 224. **C** ResNet101 model used in this study contains a total of 33 bottlenecks with a sample input image of size 3 × 224 × 224. **D** Swin V2-B model used in this study containing a total of 24 SwinTransformerBlock V2 modules and 3 PatchMergingV2 modules, with a sample input image of size 3 × 256 × 256.

Transfer learning was performed using four models pre-trained on ImageNet which is a broad image classification dataset containing over one million images across 1000 classes^78^. Fine-tuning of pre-trained model parameters was performed for each model, including EfficientNetV2-S, MobileNetV3-L, ResNet101, and Swin Transformer V2, to classify nematodes. Following the pre-trained backbones of each model, a new classification head was designed based on the seven classes. The classification head consisted of a fully connected (FC) layer followed by a LayerNorm, ReLU activation, dropout, and an FC layer with 7 units representing the logits for seven nematode species. These logits can then have the softmax operation applied to convert them to a probability distribution indicating the predictions of the model for each class. Architectures of fine-tuned models were illustrated in Fig. 5 based on LaTeX code from https://github.com/HarisIqbal88/PlotNeuralNet.

### EfficientNetV2

EfficientNetV2 is a convolutional neural network designed to improve both training speed and parameter efficiency. The model uses Fused-MBConv layers in its early stages, which combines depthwise and regular convolutions to enhance computational performance on modern hardware. In later layers of the model, regular MBConv layers, which contain depthwise convolutions, are used to decrease the computational requirement in training. EfficientNetV2 employs a non-uniform scaling strategy which selectively increases the complexity of specific layers rather than scaling all layers equally. Additionally, the model implements progressive learning which gradually increases image size and adjusts regularization during training to maintain accuracy while accelerating the process. These design selections make EfficientNetV2 more efficient and accurate than EfficientNetV1, particularly for large-scale image classification tasks^48^. We used the EfficientNetV2-Small architecture (referred to as EfficientNetV2-S in this document). The architecture of this model contains a block of a 3 × 3 convolution followed by groups of 2, 4, and 4, 3 × 3 Fused-MBConv modules. These are followed by groups of 6, 9, and 15, 3 × 3 MBConv modules which lead into the final 1 × 1 convolutional layer, pooling layer, and classification head (Fig. 5A).

### MobileNetV3

MobileNetV3 is an efficient neural network designed for mobile devices, building on the advancements of MobileNetV1 and V2. It introduces depthwise separable convolutions, which apply filters per input channel, significantly reducing computational cost and model size in CNNs. Additionally, it incorporates inverted residuals with linear bottlenecks to further optimize performance and efficiency^79^. A key feature is the use of Squeeze-and-Excite (SE) where “Squeeze,” captures global spatial information through global average pooling, and “Excite,” learns channel-wise dependencies using fully connected layers to generate weights for each channel which enhances important features while suppressing less useful ones^80^. Overall, SE layers improve the focus of the model on important features. Additionally, MobileNetV3 replaces the costly swish activation with the more efficient hard-swish. The structure of the model is refined through Neural Architecture Search^81^ which are advanced methods used to automatically discover efficient architectures for deep learning models to find the optimized model. MobileNetV3 comes in two variants including, Large and Small, which are designed for high-resource and low-resource tasks, respectively. These variants offer a balance between accuracy and speed^52^, as increasing the model size (i.e., adding more parameters) typically improves accuracy but at the cost of reduced speed. We used the MobileNetV3-Large architecture (referred to as MobileNetV3-L in this document). This model architecture contained a 3 × 3 convolutional layer followed by 15 inverted residual layers with a mixture of 3 × 3 and 5 × 5 kernels, a 1 × 1 convolutional layer, the pooling layer, and the classification head (Fig. 5B).

### ResNet101

The ResNet architecture, introduced by Microsoft in 2015, significantly advanced the field of deep learning by addressing the challenges associated with training very deep neural networks. Increasing the number of layers often led to problems such as vanishing and exploding gradients, which hindered the ability of the network to learn effectively. ResNet model architectures overcame these issues by introducing the concept of “residual blocks”. This innovation leveraged skip connections, which link the activations of a given layer to layers further down the network by bypassing intermediate layers. The primary benefit of these skip connections is their ability to mitigate the negative impact of any layer that might degrade the performance of the model. By allowing the network to bypass such layers, ResNet facilitates the training of much deeper architectures without facing traditional issues such as vanishing or exploding gradients^53^. In ResNet-101, the network consists of 101 layers. Figure 5C represents the ResNet 101 architecture used in this study. The 101-layer variant contains a convolutional layer followed by a MaxPool layer and 33 total residual bottleneck blocks, each containing three convolutional layers, totaling 101 layers. This backbone is then fed into the pooling layer and classification head.

### Swin V2

The Swin Transformer, introduced in 2021, is a novel vision Transformer. The model is designed to address the challenges of adapting Transformer architectures from language processing to vision tasks. This is achieved by using a hierarchical structure where representations are computed using shifted windows, enabling more efficient and scalable processing of visual information^82^. Building upon this foundation, the Swin Transformer V2 introduces several enhancements to overcome challenges such as training instability, resolution transfer, and dependency on large labeled datasets. To improve training stability, Swin V2 implements Residual Post-Normalization, applying normalization at the end of each layer instead of at the beginning, along with scaled cosine attention to maintain stability as the model scales. Additionally, it employs a log-spaced approach to adapt to varying resolutions, particularly aiding the transition from low-resolution training to high-resolution fine-tuning. Furthermore, Swin V2 incorporates SimMIM, a technique that reduces reliance on extensive labeled datasets while maintaining high performance^49^. In this study, we used the Swin V2-Base model (referred to as Swin V2-B in this document). The base model contains the image partitioning layer followed by two SwinTransformerBlock V2 modules. Proceeding this, there is a PatchMergingV2 block, two SwinTransformerBlock V2 modules, another PatchMergingV2 block, 18 SwinTransformerBlock V2 modules, the final PatchMergingV2 layer, and the final two SwinTransformerBlockV2 modules. The result is then passed into the pooling layer and classification head (Fig. 5D).

### Hyperparameter tuning

In order to select robust hyperparameters, a process of hyperparameter search on a pre-set search space was used. Considering that it is not feasible to search through every combination of possible hyperparameters, especially for continuous values such as the learning rate, we employed BOHB, a combination of the BO and HB algorithms^56^. This technique allowed for the search for optimal hyperparameters in the least number of training epochs. HB operates by assigning budgets to each hyperparameter configuration and only allocates additional training budgets to configurations that perform better. BO uses an objective function to iteratively improve the current configuration. BOHB combines the advantages of both algorithms by having budget al.locations for each configuration while using BO to “guide the search” instead of the random sampling used by HB^56^.

In this study, the Ray Tune implementation of BOHB was used which involves the TuneBOHB search algorithm for guided hyperparameter sampling and the HyperBandForBOHB scheduler for scheduling and pruning trials efficiently^83^. The default parameters for the BOHB implementation in Ray Tune with a reduction factor of three and a max epoch budget of 81 were used. To balance computational cost and obtain optimal results, 25 combinations of hyperparameters (or trials) were tested. Each trial could end after 1, 3, 9, 27, or 81 epochs, as determined by BOHB, depending on the relative performance of the trial compared to other trials. The best-performing trial was allowed to continue for 81 epochs. The hyperparameter search space was identical for all models.

The tuned hyperparameters included the learning rate, which controlled the optimization steps, and was set within a range from 10⁻⁵ to 10⁻³ using a log-uniform distribution. The dropout rate, aimed at preventing overfitting, was tuned within an interval of 0 to 0.5 using a uniform distribution. To enhance data variability, maximum random rotation and brightness adjustments were set between 0° to 30° and 0 to 0.1, respectively, with uniform distributions. The size of the FC layer in the classification head, which balances complexity and performance, was chosen from the integer interval of 256 to 1024 using a discrete uniform distribution. Batch size, determining the number of samples processed per update, was either 32 or 64 for each model. The best epoch was selected based on balanced validation accuracy.

### Fine tuning

During each trial, fine-tuning was performed using gradual unfreezing which occurred over five training epochs. Gradual unfreezing is a fine-tuning process that starts the training with an unfrozen top layer while keeping the weights of the remaining lower layers constant (frozen) during training, allowing the model to retain pre-trained knowledge to use on a new task^69^. In subsequent epochs, the topmost frozen layers, and eventually the whole model, are unfrozen. In this study, gradual unfreezing for all hyperparameter configurations started if the configuration reached 50% balanced validation accuracy during training. This threshold ensures that the pre-trained weights are effectively utilized for the nematode classification task before being modified. Given the varying number of layers across the four pre-trained model architectures, gradual unfreezing was applied over five epochs. During this process, approximately 20% of the model is unfrozen per epoch, allowing a progressive fine-tuning of the layers. By the end of the five epochs, the entire model is unfrozen and fully available for training. This process ensures that all layers are gradually unfrozen from top to bottom.

### Model training

The pretrained weights for each of the EfficientNetV2-S, MobileNetV3-L, ResNet101, and Swin V2-B models were provided by Torchvision (https://github.com/pytorch/vision). The modified classification heads were attached. The classification head was added to these backbones using standard PyTorch modules^84^. In addition, the training and evaluation processes were written with PyTorch. In the model training phase, image preprocessing and augmentation were used on the inputs of the model. In addition, after each batch ended, the model parameters were updated according to the AdamW optimizer. At the end of each epoch, the model was set to the “evaluation phase” to calculate the evaluation metrics. The models were trained on the University of Maryland Zaratan HPC Cluster using an NVIDIA A100 40GB GPU. To enhance model training and evaluation speed on this GPU, automatic mixed precision of PyTorch was used.

### Model evaluation metrics

The performance of models was evaluated using fitness metrics including balanced accuracy, macro precision, ROC-AUC (receiver operating characteristic - area under the curve), macro OVO ROC-AUC score, macro F1-score, macro specificity (true negative rate) (Eqs. 1–5). Accuracy is defined as the proportion of correctly classified instances, where true positives (TP) and true negatives (TN) are correctly identified, while false positives (FP) and false negatives (FN) are misclassified instances. Precision measures the proportion of true positives out of all positive predictions, while specificity assesses the correct classification of negative instances. The F1-score is the harmonic mean of precision and recall (true positive rate), providing a balanced measure when precision and recall differ. Furthermore, macro metrics are calculated by averaging the metric across all classes, giving equal weight to each class, regardless of the number of observations in each class. Balanced accuracy, macro precision, macro F1-score, and macro OVO ROC-AUC were calculated using scikit-learn^85^, while macro specificity was calculated using imbalanced-learn^86^.

The following equations define the parameters.

1 $${\text{Balanced}}\;{\text{accuracy }}=\frac{1}{K}\mathop \sum \limits_{{i={\text{1}}}}^{K} \frac{{T{P_i}}}{{T{P_i}+F{N_i}}}$$

2 $${\text{Macro}}\;{\text{Precision}}=\frac{1}{K}\mathop \sum \limits_{{i=1}}^{K} \frac{{T{P_i}}}{{T{P_i}+F{P_i}}}$$

3 $${\text{Macro}}\;{\text{OVO}}\;{\text{ROC-AUC}}\;{\text{Score}}=\frac{2}{{K\left( {K - 1} \right)}}\mathop \sum \limits_{{i=1}}^{K} \mathop \sum \limits_{{j=i+1}}^{K} \frac{1}{2}\left( {\mathop \int \limits_{0}^{1} T{P_i}dF{P_i}+\mathop \int \limits_{0}^{1} T{P_j}dF{P_j}} \right)$$

4 $${\text{Macro}}\;{\text{F1-Score}}=\frac{1}{K}\mathop \sum \limits_{{i=1}}^{K} \frac{{2T{P_i}}}{{2T{P_i}+F{P_i}+F{N_i}}}$$

5 $${\text{Macro}}\;{\text{specificity}}=\frac{1}{K}\mathop \sum \limits_{{i=1}}^{K} \frac{{T{N_i}}}{{T{N_i}+F{P_i}}}$$

where *TP*_*i*_, *TN*_*i*_, *FP*_*i*_, and *FN*_*i*_ represent true positives, true negatives, false positives, and false negatives for class *i* respectively, and *K* indicates the number of classes.

### Dataset II validation

To verify the performance of the models on a similar end-user platform, a total of 77 new images were collected including 13 *Helicotylenchus*, 10 *Hoplolaimus*, 14 *Pratylenchus*, 4 *Meloidogyne*, 4 *Mesocriconema*, 18 *Trichodorus*, and 14 *Tylenchorhynchus* which is referred to as dataset II. Additional validation data were obtained with the inverted microscope setup, similar to the training video data acquisition described above, but captured as individual images with 10× and 20× microscope objectives.

An image classification web application was created using Gradio^87^. This web application allowed for cropping and classification of an image or a batch of images all within the application. The images were automatically cropped using the method described in the dataset preprocessing section. In case of automatic cropping failure, manual cropping was needed. The probability predictions of models for each image were displayed on the application and saved. The metrics were calculated using the same process mentioned for model evaluation.

### Model demonstration

An additional validation step was conducted to determine the generalizability of the models. EfficientNetV2-S was the best-performing model based on the reported metrics of macro precision, ROC-AUC, macro F1-scores, and macro specificity. The models were incorporated into the Gradio application hosted on Hugging Face Spaces which can be accessed at https://huggingface.co/spaces/VikramR/NematodeClassifier. This application allows for the classification of images containing a single nematode belonging to one of the 7 genera used by the model and is available for public use. In addition, the automatic cropping pipeline was included for testing with the new set of microscope images that were not cropped. A similar effort has been reported in other studies focusing on nematode and plant disease classification^20,31,88^, where model reliability and performance have been validated on user interfaces.

## Supplementary Information

Below is the link to the electronic supplementary material.

Supplementary Material 1

Supplementary Material 2

## Data Availability

All project code is publicly available at [https://doi.org/10.5281/zenodo.16271445](https:/doi.org/10.5281/zenodo.16271445)Nematode image data are available through Ag Data Commons: [https://doi.org/10.15482/USDA.ADC/27244674](https:/doi.org/10.15482/USDA.ADC/27244674).

## Code availability

All project code is publicly available at 10.5281/zenodo.16271445.
