# Supplementary material for: Identification of plant-parasitic nematode genera in turfgrass using deep learning algorithms
Source: Sci Rep. 2025 Dec 7;16:24. doi: 10.1038/s41598-025-29467-4 (PMC12764819; doi:10.1038/s41598-025-29467-4)
Supplement: Supplementary file 1 — Supplementary Material 1 [file 41598_2025_29467_MOESM1_ESM.docx]

Table S1. Statistical fitness metrics (%) of EfficientNetV2-S, MobileNetV3-L, ResNet101, and Swin V2-B on the training and validation data.

| Model architecture | Training balanced accuracy | Training macro precision | Training macro OVO ROC-AUC score | Training macro F1-score | Training macro specificity | Validation balanced accuracy | Validation macro precision | Validation macro OVO ROC-AUC score | Validation macro F1-score | Validation macro specificity |
| --- | --- | --- | --- | --- | --- | --- | --- | --- | --- | --- |
| EfficientNetV2-S | 99.97 | 99.98 | 100.00 | 99.97 | 100.00 | 96.25 | 95.73 | 99.88 | 95.91 | 99.33 |
| MobileNetV3-L | 97.82 | 97.27 | 99.96 | 97.52 | 99.54 | 91.22 | 90.39 | 99.39 | 90.37 | 98.45 |
| ResNet101 | 99.86 | 99.86 | 99.99 | 99.86 | 99.97 | 89.59 | 90.87 | 98.93 | 89.46 | 98.41 |
| Swin V2-B | 99.65 | 99.34 | 99.99 | 99.49 | 99.91 | 94.25 | 94.26 | 99.68 | 94.19 | 99.09 |

OVO ROC-AUC = one versus one receiver operating characteristic - area under the curve

Table S2. Hyperparameters of Swin V2-B model with a batch size of 32.

| Best training epoch | Learning rate | Dropout rate | Maximum random rotation | Maximum random brightness | FC layer units in classification head | Batch size |
| --- | --- | --- | --- | --- | --- | --- |
| 75 | 1.77×10^-4^ | 25.09% | 12.44 | 0.0944 | 921 | 32 |

FC = fully connected

Table S3. Statistical fitness metrics (%) of Swin V2-B with a batch size of 32 on the test dataset.

| Balanced accuracy | Macro precision | Macro OVO test ROC-AUC score | Macro F1-score | Macro specificity |
| --- | --- | --- | --- | --- |
| 92.79 | 91.65 | 99.46 | 92.07 | 98.73 |

OVO ROC-AUC = one versus one receiver operating characteristic - area under the curve

Table S4. Statistical fitness metrics (%) of EfficientNetV2-S, MobileNetV3-L, ResNet101, and Swin V2-B on the additional validation dataset.

| Model architecture | Balanced accuracy | Macro precision | Macro OVO ROC-AUC score | Macro F1-score | Macro specificity |
| --- | --- | --- | --- | --- | --- |
| EfficientNetV2-S | 82.47 | 83.56 | 96.70 | 80.02 | 96.55 |
| MobileNetV3-L | 54.35 | 60.45 | 87.01 | 52.70 | 91.37 |
| ResNet101 | 68.94 | 71.57 | 89.93 | 68.29 | 93.99 |
| Swin V2-B (batch size 64) | 73.68 | 68.41 | 92.36 | 67.65 | 94.25 |
| Swin V2-B (batch size 32) | 71.70 | 74.65 | 93.91 | 67.57 | 94.08 |

OVO ROC-AUC = one versus one receiver operating characteristic - area under the curve
