# Supplementary material for: Identification of plant-parasitic nematode genera in turfgrass using deep learning algorithms
Source: Sci Rep. 2025 Dec 7;16:24. doi: 10.1038/s41598-025-29467-4 (PMC12764819; doi:10.1038/s41598-025-29467-4)
Supplement: Supplementary file 2 — Supplementary Material 2 [file 41598_2025_29467_MOESM2_ESM.docx]

**Algorithm 1: Frame Extraction**

- Let V = Set of 1887 nematode videos
- For each video:
  - Extract every 100 ^th^ frame
  - Crop out black bars on edges of these frames
  - Save to disk, keeping track of the specimen from which the nematode came from, magnification level, and nematode species, and frame index

**Algorithm 2: Frame Deduplication**

- Let R = The 31,684 raw frames produced by the Frame Extraction algorithm
- For each of the 3 magnification levels (10×, 20×, 40×):
  - Let F = Subset of R with the specified magnification level
  - For each of the 7 nematode classes:
    - Let F_2_ = Subset of F_1_ with images of the specified nematode class
    - Let G = A partition of F_2_, with each subset of frames belonging to a single specimen
    - For each set of frames F_3_ in G:
      - Sort F_3_ in terms of each frame’s time in its original video (0, 100, 200, etc.)
      - Let I = Empty list of cropped images
      - Let M = Empty list of binary masks for the associated element of I
      - For each frame f in F_3_:
        - Let i, m = CROP_FRAME(f)
        - Add i to I, and add m to M
      - Let prev_img = M[0], first frame mask in M
      - Let deduplicated_frames = [I[0]], list containing first frame in I
      - For each image-mask pair (i, m) in (I, M):
        - Let sim = % agreement between prev_img, m
        - If sim < 80%:

Add i to deduplicated_frames

Set prev_img = m

- - - - For each deduplicated frame d in deduplicated_frames:
        - Saved to the disk

**Algorithm 3: CROP_FRAME**

- Let frame = provided parameter, RGB frame of uncropped nematode frame
- Let gray = frame converted to grayscale
- Let kernel = 11 by 11 matrix filled with 1
- Let edges = Canny edge detection of gray
- Let edges_dilate = morphological dilation of edges using kernel with 3 iterations
- Let edges_erode = morphological erosion of edges_dilate using kernel with 3 iterations
- Let contour = Largest contour within edges_erode, representing the nematode position in a binary image mask
- Let (x_1_, y_1_, x_2_, y_2_) = Bounding box of the nematode within contour, which can be found using the argmax operator
- Let cropped_frame = frame cropped to bounding box (x_1_, y_1_, x_2_, y_2_)
- Let cropped_mask = contour cropped to bounding box (x_1_, y_1_, x_2_, y_2_)
- Resize cropped_mask to 224 by 224
- Return cropped_frame, cropped_mask
